# Suicidality Assessment of the Elderly With Physical Illness in the Emergency Department

**DOI:** 10.3389/fpsyt.2020.558974

**Published:** 2020-09-11

**Authors:** Alessandra Costanza, Andrea Amerio, Michalina Radomska, Julia Ambrosetti, Sarah Di Marco, Massimo Prelati, Andrea Aguglia, Gianluca Serafini, Mario Amore, Guido Bondolfi, Laurent Michaud, Maurizio Pompili

**Affiliations:** ^1^ Department of Psychiatry, Faculty of Medicine, University of Geneva (UNIGE), Geneva, Switzerland; ^2^ Department of Psychiatry, ASO Santi Antonio e Biagio e Cesare Arrigo Hospital, Alessandria, Italy; ^3^ Section of Psychiatry, Department of Neuroscience, Rehabilitation, Ophthalmology, Genetics, Maternal and Child Health, University of Genoa, Genoa, Italy; ^4^ Department of Psychiatry, IRCCS Ospedale Policlinico San Martino, Genoa, Italy; ^5^ Mood Disorders Program, Tufts Medical Center, Boston, MA, United States; ^6^ Faculty of Psychology, University of Geneva (UNIGE), Geneva, Switzerland; ^7^ Emergency Psychiatric Unit, Department of Psychiatry and Emergency Department, Geneva University Hospitals, Geneva, Switzerland; ^8^ Department of Psychiatry, Service of Liaison Psychiatry and Crisis Intervention, Geneva University Hospitals, Geneva, Switzerland; ^9^ Department of Psychiatry, Lausanne University Hospital, Lausanne, Switzerland; ^10^ McGill Group for Suicide Studies, McGill University, Montreal, QC, Canada; ^11^ Department of Neurosciences, Mental Health and Sensory Organs, Suicide Prevention Center, Sant’Andrea Hospital, Sapienza University of Rome, Rome, Italy

**Keywords:** suicide, suicidal behavior, suicide attempt, suicidal ideation, elderly, physical illness, risk factors, emergency department

## Introduction

Taking into account and evaluating the presence of a physical illness plays a crucial role in the clinical encounter with the elderly who may present suicidal ideation (SI) and suicidal behavior (SB) (1, 2).

On the one hand, physical illness is associated with greater suicidality risk in the elderly. This association has been inferred from both quantitative and qualitative findings based on population and registry cohorts (3–5), case-control studies (6–13), psychological autopsies (14, 15), coroners’ reports (16, 17), and suicide notes (17, 18) [for reviews, see (19, 20)]. This applies to SI/wishes to die (20–22) and the entire span of SB, including suicide attempts (SAs) and completed suicides [for reviews, see (20, 23, 24)].

On the other hand, a physical illness may render the suicidality assessment of the elderly complex for multiple reasons (25): a) the possible presence of uncommon or masking clinical features of both SB (indirect or passive SB, e.g. self-starvation) and psychiatric disorders associated with SB (e.g. atypical depressive disorders with prevalent somatic or cognitive symptoms) (26–28); b) the risk of overlooking and missing SB when severe illnesses coexist (29); c) the frequent reticence among the elderly in externalizing SI as they place more emphasis on their physical conditions (30–33); and d) the eventual caregivers’ representations of suicide as a more “understandable” act when facing greater physical fragilities and the intrinsic proximity of the end of life (34, 35). S. de Beauvoir wrote in the 1970s about the feeling of resignation or impotence of what may be considered an inexorable outcome: “Some suicides of elder people follow states of neurotic depression that one has not been able to heal; but most are normal reactions to an irreversible, desperate situation, experienced as intolerable” (36).

A large number of the elderly who died by suicide had had recent contact with primary healthcare professionals, including in emergency departments (EDs). Approximately 50 to 70% of individuals had consulted a healthcare professional in the 30 days preceding their death (32, 37), and more than 80% had done so in the six months prior to death (38). In most of these cases, the last consultation had focused on physical complaints in the absence of a psychiatric diagnosis (32, 37). Notably, affective disorders in the geriatric population can go undiagnosed by ED physicians (39).

The aim of this opinion paper is to point out the opportunity of assessing suicidality in the elderly when they present to the ED with physical illness. To this purpose, it could be useful to overview some both controversial and consensual key points on suicidality risk in the elderly, as discussed below.

## Discussion

### Some Controversial Matters

When considering whether the presence of the physical disease is a significant risk factor for suicidality in older versus younger patients, a legitimate objection could be that the former has statistically higher somatic susceptibility. The same objection could be raised to the argument that an the increasing rate of SI/wishes to die (21), SAs (40), and completed suicides (5, 6, 8, 41) in the elderly has been observed in the presence of multiple somatic illnesses (a “burden of physical illness”). The answer to this question is probably addressed by qualitative studies, from which the subjective attributions of mental suffering as a consequence of one or more physical illnesses have emerged (14, 15, 17, 18).

Another debated point is the extent to which mental comorbidities contribute to the suicidality risk in the elderly (1). Psychiatric disorders such as major depressive disorder and substance use disorder have been shown to play a significant role in death by suicide among individuals older than 65 years (6, 42, 43). While some studies found that the effect of physical illness on suicidality risk persisted even after controlling for comorbid mental disorders (5, 9), others have relegated physical illness to a secondary contributing risk factor (16, 29). Major depressive disorder, in analogy with functional impairment or pain, could be considered as a possible mediating factor that partially explains the link between physical illness and suicidality risk (physical illness causes/contributes to the occurrence of depressive disease and the latter increases suicidality risk); similarly, substance use disorder (e.g., alcohol, benzodiazepine, or opioids abuse) may be included in this reciprocal link, initially interpreted as tentative of self-medication that eventually exacerbates both major depression and suicidality (4, 19, 28, 43–45).

### And Some Common Clinical Features**

In recent years, studies have highlighted the role of physical illness, especially among the oldest patients. Physical illness exerted a stronger motivational effect for suicide in old-old (≥75 years old) attempters compared to their young-old (65–74 years old) and middle-aged (64–50 years old) counterparts (46). One-third of those 70+ years of age who had attempted suicide attributed their act to somatic distress (47). Among those who had died by suicide, a greater incidence of physical illness was reported in the old-old compared to the young-old (38, 48) and middle-aged adults (38). Those in whom the reason for completed suicide was attributed to the presence of physical illness were older than those in whom the reason was attributed to the presence of mental illness (17). Hospitalization due to physical illness had the greatest influence on the risk of completed suicide among the old-old (41).

Contrary to findings in the general population, in the elderly non-lethal events seem to be more common in males (1), especially among the young-old where this has been attributed to so-called “elderly adolescentism” (49). Improvements to welfare and healthcare may have led to a rejuvenation of the 65–74 age group, which could be at the origin of certain behavioral patterns such as SA intended as a “cry-for-help” in response to environmental adversities (49). In this case, the stressful context could be represented by the occurrence of one or more physical illnesses (49). In studies that did not utilize the distinction between old-old, middle-aged, and young adults, the proportion reporting that SA was due to physical illness did not differ between males and females in the 70+ age group (47, 50). As far as completed suicides, the presence of physical illness should be considered as a warning sign, especially in males (15), in particular, those with serious and multiple illnesses (6). The risk of completed suicides has been shown to differ between males than females depending on the type of physical illness (4). In the old-old patients, hospitalization with a physical illness conferred a greater risk of completed suicides in males (41).

Globally, neurological diseases, pain, and oncological conditions occurred more frequently in the suicidal elderly. An association between neurologic diseases and SI, SA, and SB was observed (6, 12, 51–55), especially for stroke and hemiplegia (4, 11, 13, 56, 57), epilepsy (4, 8, 45, 58), and dementia (13, 59, 60). A greater rate of SI was documented in patients with Parkinson’s disease (60, 61), and the role of sub-thalamic deep brain stimulation (DBS) on suicidality risk in patients treated for extrapyramidal movement disorders is still discussed [for a recent systematic review, see (62)]. The pain was significantly and independently associated with SI/wishes to die (21, 22, 63–66) and completed suicides (8, 10, 17). Oncological conditions in the elderly were shown to be associated with SI, and the entire span of SB (3, 4, 6, 7, 11, 13, 17, 67, 68).

## Conclusions

The elderly who attend the ED with a physical illness are vulnerable individuals and the ED visit often represents a “sentinel event” that may signal a medical or psychosocial fracture in their established equilibrium (69, 70).

In addition to investigation and management of physical illness, attention needs to be paid to its psychic repercussion on the elderly. This also includes addressing and assessing suicidality that, for the reasons synthesized in the introduction, is frequent in this population but can be missed by the clinician. In a specular way, recommendations on suicidality prevention measures in the elderly encourage a so-called “multi-faceted” approach, which emphasizes the in-depth consideration of aspects related to the presence of physical illness, considered among the most relevant determinants of the elderly’s SI and SB (37, 71–74).

This opportunity involves both primary healthcare professionals and psychiatrists. The ED represents a clinical setting where the elderly with both physical illness and greater suicidality risk frequently converge. Conversely, the ED, by offering an integrated somatic/psychiatric approach, constitutes a precious resource for this complex and fragile population.

**Figure 1:**
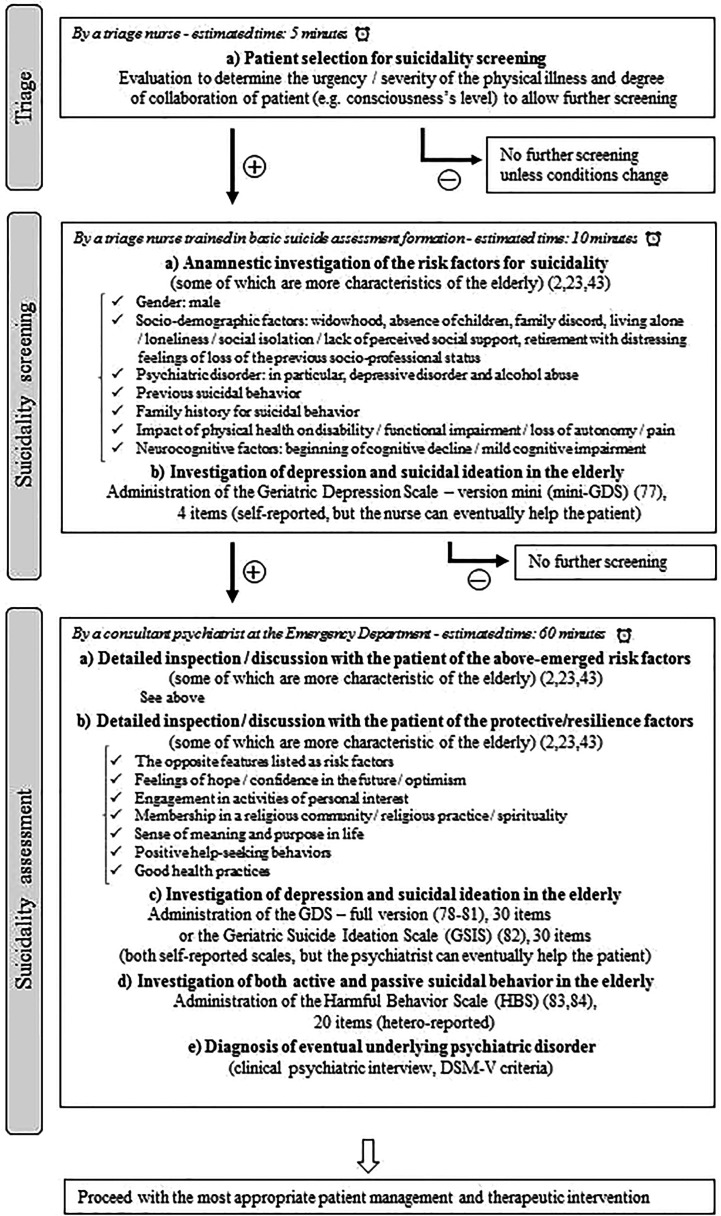
A proposal for a potential tiered suicidality assessment in the elderly with physical illness attending an Emergency Department.

Not every elderly patient who arrives at the ED with physical illness can be screened for suicide. Thus, there are some pragmatic considerations, which would limit this approach in the clinical practice. They are dictated by the clinical condition of the patient (e.g. urgency/severity of the physical illness, consciousness’s level) and the amount of resources, regarding both staff and time, which can be allotted for the suicidality assessment. To achieve a more balanced cost-benefit ratio, we propose —mainly on the basis of a previous Canadian work (75)— a potential example of a tiered assessment (2, 23, 43, 76–83) **(** **Figure 1** **)**.

The ED, the place of what cannot be deferred, may be finally at the center of the clinical and human encounter with the elderly who, confronted with the possibility of approaching the end of their existences (perceived as more concrete or urgent by the presence of physical illness), present a moral pain experienced as non-repairable. The dialogue with these patients in the ED can constitute the beginning of a therapeutic relationship aimed at trying to understand the individual meaning to the urgency of their days, and therefore to explore an alternative to suicide as unique possibility to avoid the unbearable psychache.

Future research is needed to refine the comprehension of the suicidality peculiarities in the elderly population and translate it into clinical practice through an eventual feasible, validated, and consensual screening.

## Author Contributions

AC, AAm, MR, and JA researched the literature and drafted the primary manuscript. SM, MPr, AAg, and GS carefully revised the manuscript. GS, MA, GB, LM, and MPo supervised all steps of the work and provided the intellectual impetus. All authors contributed to the article and approved the submitted version.

## Conflict of Interest

The authors declare that the research was conducted in the absence of any commercial or financial relationships that could be construed as a potential conflict of interest.
